# Effect of *Lachnum* YM156 Polyphenol on STAT3/COX-2 Signal Pathway and Gut Microbiota in Mice with N-Nitrosodiethylamine-Induced Hepatic Injury

**DOI:** 10.17113/ftb.63.04.25.8809

**Published:** 2025-12-26

**Authors:** Tingting Chen, Dong Liu, Ming Ye

**Affiliations:** 1 School of Food and Biological Engineering, Hefei University of Technology, No. 193, Tunxi Road, Hefei City, Postal Code 230009, PR China; 2 Anhui Zhanshi Food Corporation, No. 6, South Outer Ring Road, Ningguo City, Postal Code 242300, PR China

**Keywords:** *Lachnum* polyphenol, STAT3/COX-2 signalling pathway, gut microbiota, liver injury

## Abstract

**Research background:**

Global food security faces increasing threats from chemical contaminants, with N-nitrosodiethylamine (NDEA) emerging as a potent hepatotoxicant of significant concern. NDEA-induced hepatic injury causes a pathological triad: (*i*) reactive oxygen species-mediated oxidative cascades, (*ii*) nuclear factor κB-driven inflammatory amplification, and (*iii*) gut microbiota-derived endotoxin translocation. Although natural polyphenols have established protective efficacy, fungus-derived variants remain pharmacologically enigmatic, particularly regarding their pathway-specific regulation and microbiota modulation. We comprehensively investigated the therapeutic capacity of *Lachnum* polyphenols for hepatoprotection.

**Experimental approach:**

The hepatoprotective and microbiota-modulating efficacy of extracellular polyphenols from *Lachnum* YM156 (LSP156) was evaluated in an NDEA-induced mouse model. Sixty male Institute of Cancer Research (ICR) mice were randomised into six experimental groups receiving 28-day oral LSP156 treatment. Body mass measurements, hepatosomatic indices, systemic oxidative stress biomarkers (superoxide dismutase [SOD] and malondialdehyde) and proinflammatory cytokines (interleukin [IL]-6 and tumour necrosis factor [TNF]-α) were assessed. Hepatic histopathology was analysed by haemotoxylin and eosin staining, whereas immunoblotting with chemiluminescence detection assessed the STAT3/COX-2 pathway activation. Gut microbiota composition was profiled through 16S rRNA sequencing.

**Results and conclusions:**

After 28-day oral administration (50-100 (mg/kg)/day), LSP156 significantly improved somatic growth parameters (body mass gain) and organ indices in NDEA-induced mice. LSP156 increased the activities of SOD and catalase, as well as glutathione levels, and greatly reduced the liver function markers alanine aminotransferase, aspartate aminotransferase, alkaline phosphatase and total bilirubin. It also improved liver cell damage in tissue samples compared to model controls. LSP156 halted the activation of STAT3 and reduced TLR4 levels, which lowered cyclooxygenase protein levels and protected the liver from damage. LSP156 enhanced the digestion and absorption of carbohydrates and proteins, as well as the biosynthesis of terpenoids such as ubiquinone in mice, by rectifying intestinal flora imbalances, modifying the flora structure and demonstrating a strong correlation between *Bacteroidales* and *Lactobacillales* with the reduction of TNF-α and IL-6. The LSP156 demonstrated dose-dependent therapeutic efficacy in attenuating oxidative stress, hepatocyte impairment and systemic inflammation.

**Novelty and scientific contribution:**

Fungal polyphenol LSP156 maintains balanced gut bacteria by simultaneously managing inflammation and oxidation. These findings suggest a new approach to designing drugs that target multiple factors in complex metabolic disorders.

## INTRODUCTION

N-nitrosodiethylamine (NDEA), one of the seven N-nitrosamines, is found in food, and is potentially toxic. Nitrite, which is used as a preservative, produces N-nitrosamines in food (*1*-*5*). NDEA can induce various diseases in mice, including renal injury (*6*), metabolic disturbances (*7*), liver injury (*8*) and liver cancer (*9*), and is classified as a 2A carcinogen (probably carcinogenic to humans) by the International Agency for Research on Cancer (*10*). Cell damage from oxidative stress and free radicals is recognised as a key cause of NDEA toxicity. The free radicals are NDEA metabolites produced by cytochrome P450 families *in vivo*, with the primary metabolic sites in the liver (*11*, *12*).

The gut microbiota is influenced by dietary habits, lifestyle, drugs, and other environmental factors and is also regulated by genetic factors (*13*). The development of culture-independent sequencing methods, such as 16S rRNA gene profiling and shotgun metagenomics, has revolutionised high-throughput characterisation of complex microbial ecosystems (*14*). The gut-liver axis forms a pivotal interface in hepatointestinal pathophysiology, with dual mechanistic roles. Functioning as both a primary immune organ for systemic bacterial clearance and a metabolic nexus for lipid homeostasis, the liver maintains direct anatomical connectivity with the intestinal tract through portal circulation (*15*). Intestinal mucosal immunity regulates innate and adaptive responses, while hepatic tissue serves as the initial extraintestinal site processing enteric microbial metabolites and portal venous outflow from both colonic and ileal regions (*16*, *17*). Gong *et al.* (*16*) reported that the gut microbiota influenced the diurnal variation of acetaminophen-induced acute liver injury. Furthermore, the structure of the gut microbiota plays a role in systemic inflammation in patients with cirrhosis and non-alcoholic fatty liver disease (NAFLD) (*18*).

Polyphenols, natural metabolites extracted from vegetables, fruits and microorganisms, are obtained through the daily diet, and their strong antioxidant and free radical scavenging properties play an important role in the food industry (*19*). Researchers found that polyphenols derived from *Lachnum* sp. have antioxidant and anticoagulant properties (*19*, *20*). We evaluated the therapeutic effect of LSP156 polyphenols on liver injury and their possible mechanism.

## MATERIALS AND METHODS

### Materials and reagents

The *Lachnum* YM156 strain was preserved at the Microbial Resource and Application Research Centre of Hefei University of Technology. Mycelium was obtained by fermentation using liquid potato dextrose agar medium, and polyphenols were extracted using a modified protocol (*20*). The fermented mycelium was collected and mechanically homogenised. It was extracted with 70 % ethanol (Tianjing Zhiyuan Chemical Reagent Co., Ltd., Tianjing, PR China) at a ratio of 1:10 for 4 h. The polyphenol extract was concentrated by rotary distillation at 45 °C for 2 to 3 h. We purified the crude extract using XAD-4 macroporous resin (Rohm & Haas, Philadelphia, PA, USA) under the following conditions: concentration 10 mg/mL, flow rate 2 mL/min and eluent 70 % ethanol. The main fraction was dried in an FD-1B-50 lyophiliser (Beijing Bo Medical Kang Experimental Instrument Co., Ltd., Beijing, PR China) to obtain LSP156.

ELISA kits (Nanjing Jiancheng Biotechnology Co., Ltd., Nanjing, PR China) were used to quantify interleukin-6 (IL-6), IL-1 and tumour necrosis factor-α (TNF-α). Test kits for assessing antioxidant glutathione (GSH), catalase (CAT), superoxide dismutase (SOD), malondialdehyde (MDA) and liver injury markers (alanine aminotransferase [ALT], aspartate transaminase [AST], total bilirubin [TBiL], and alkaline phosphatase [AKP]) were also provided by Nanjing Jiancheng Biotechnology Co., Ltd.

### Animal model preparation

All experimental procedures adhered to the Institutional Animal Care and Use Committee guidelines for animal experimentation. Formal approval was obtained from the Laboratory Animal Welfare and Ethics Committee of Hefei University of Technology (approval no. HFTLAWEC20212008). Sixty male ICR mice (specific-pathogen-free (SPF) grade, 6–8 weeks old) were obtained from the Experimental Animal Centre of Anhui Medical University (Hefei, PR China). The animals were housed in polypropylene cages under controlled conditions ((24±1) °C, (60±5) % humidity) with *ad libitum* access to food and water, following a standardised 12-hour light/dark cycle in the animal facility of Hefei University of Technology. After a 7-day acclimatisation period, the mice were randomised into six groups (*N*=8): (*i*) vehicle control (C), (*ii*) *w*(NDEA)=100 mg/kg (M), (*iii*) *w*(NDEA)=100 mg/kg+*w*(LSP156)=50 mg/kg×4 weeks (AL), (*iv*) *w*(NDEA)=100 mg/kg+*w*(LSP156)=100 mg/kg×4 weeks (AH), (*v*) *w*(NDEA)=100 mg/kg×4 weeks followed by LSP156 50 mg/kg×4 weeks (BL), and (*vi*) *w*(NDEA)=100 mg/kg×4 weeks followed by *w*(LSP156)=100 mg/kg×4 weeks (BH). After the treatment period, stool samples were collected, and the mice were sacrificed by cervical dislocation. Blood was collected from the eyeball and centrifuged (centrifuge 5804R; Eppendorf, Hamburg, Germany) at 3000×*g* and 4 °C for 10 min to obtain serum, and the kidneys, spleen and liver were dissected and weighed. All materials were stored in a refrigerator at −80 °C.

### Measurements of physiological and biochemical indices

The manufacturer’s guidelines were followed to measure the oxidative stress parameters, GSH, CAT, SOD and MDA (Nanjing Jiancheng Biotechnology Co., Ltd) in the liver tissue homogenate. The experiments were conducted according to the guidelines provided in the kit. Tissue homogenates were prepared in ice-cold physiological saline (1:9 *m*/*V*) and centrifuged (centrifuge 5804R; Eppendorf) at 3000×*g* and 4 °C for 10 min. The supernatant was collected for analysis. Reduced GSH was quantified using the 5,5'-dithiobis-(2-nitrobenzoic acid) reagent: samples were incubated at 37 °C for 10 min in darkness, and absorbance was measured at 412 nm (752N; Shanghai Jinghua Instrument Co. Ltd., Shanghai, PR China). GSH content (nmol per mg of protein) was calculated from a standard curve. CAT activity was determined by measuring the decomposition rate of hydrogen peroxide at 240 nm. The SOD activity was assessed using the water-soluble tetrazolium salt-8 (WST-8) method at 450 nm and calculated based on the inhibition rate. MDA content was measured using thiobarbituric acid at 95 °C for 30 min, while absorbance was read at 532 and 600 nm after cooling. Total absorbance was determined using the following formula:


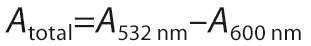
 /1/

Liver damage was evaluated by measuring serum activities of AST and ALT, and serum levels of TBil and AKP using commercial assay kits. Liver tissues were homogenised in ice-cold phosphate-buffered saline (1:9 *m*/*V*) and centrifuged (centrifuge 5810R; Eppendorf, Vienna, Austria) at 12 000×*g* and 4 °C for 15 min. The supernatant was incubated at 37 °C, and absorbance was measured at 340 nm at 1 and 2 min using an ultraviolet (UV) spectrophotometer (752N; Jinghua Science and Technology Instruments, Co., Ltd. Shanghai, PR China). Enzyme activities were calculated according to the manufacturer’s instructions.

The following formula was used to calculate the ALT activity (U per mg protein):


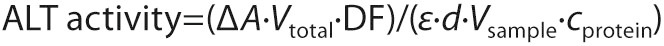
 /2/

where *V*_total_ is the total volume of the reaction solution, DF is the dilution factor, *ε* is the molar absorption coefficient of NADH (6.22·10^3^), *d* is the optical path length of the cuvette, *V*_sample_ is the volume of the sample, and *c*_protein_ is the protein concentration of the liver tissue homogenate.

A volume of 50 μL of a mixed solution containing α-ketoglutarate and aspartate was added to each reaction well, followed by the addition of 10 μL of sample. The mixture was incubated at 37 °C for 30 min. Then, 50 μL of 2,4-dinitrophenylhydrazine chromogenic solution were added and thoroughly mixed, and the reaction was allowed to proceed at 37 °C for an additional 20 min. The reaction was terminated by adding 500 μL of 0.4 mol/L NaOH, with vigorous mixing. After incubation at room temperature for 10 min, the absorbance (*A*) of both standard and sample solutions was measured at a wavelength of 510 nm (Shanghai Jinghua Instrument Ltd.). The measured absorbance values were fitted to a standard curve described by the following polynomial equation:


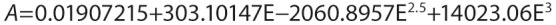
 /3/

and AST activity (U per g protein) was calculated as:


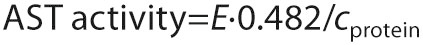
 /4/

where *E* is the enzyme activity in Karmen units, 0.482 is the conversion factor from Karmen units to U/L, and *c*_protein_ is the protein concentration in the tissue homogenate expressed in g/L.

TBil (μmol/L) was measured using the diazo method. Aliquots (50 μL) of blank (water), total bilirubin standard (85.5 μmol/L) and sample were mixed sequentially with 1.0 mL of caffeine–sodium benzoate and 0.25 mL of the diazo reagent. After a 10-minute reaction in darkness, the absorbance was read at 530 nm (Shanghai Jinghua Instrument Ltd.).


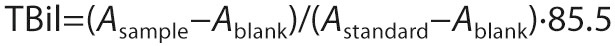
 /5/

The activity of AKP (U/L) was measured using spectrophotometry. Briefly, 20 μL of the sample were mixed with 1.0 mL of the 2-amino-2-methyl-1-propanol buffer (pH=10.4) and 200 μL of *p*-nitrophenyl phosphate (*p*NPP) substrate solution for the reaction. The absorbance was measured at 405 nm using a model 752 UV spectrophotometer (Shanghai Jinghua Instrument Ltd.).


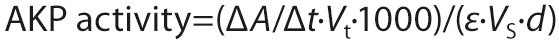
 /6/

The total reaction volume (*V*_t_) and sample volume (*V*_s_) were 310 and 10 μL, respectively. The *p*NPP molar absorption coefficient (*ε*) and optical path (*d*) were 18.5·10^3^ L/(mol·cm) and 1 cm, respectively.

Serum levels of TNF-α, IL-1 and IL-6 were quantified with commercially available enzyme-linked immunosorbent assay kits (Nanjing Jiancheng Biotechnology Co., Ltd.). The serum was isolated by centrifugation (centrifuge 5804R; Eppendorf) at 1000×*g* and 4 °C for 20 min. Then, 100 μL of the standard or serum were incubated at 37 °C for 90 min. After washing, 100 μL of the detection antibody were added and incubated at 37 °C for 60 min. Subsequently, 100 μL of the enzyme conjugate were added and incubated for 30 min at 37 °C. Next, 100 μL of the chromogen were added and developed in darkness for 15 min at 25 °C. The reaction was stopped, and the absorbance was measured at 450 nm (Multiskan GO 1510; Thermo Fisher Scientific, Vantaa, Finland).

### Histopathological assessments

Fresh liver tissues were collected immediately from different groups of mice, washed with cold normal saline, fixed in 4 % phosphate-buffered paraformaldehyde overnight, dehydrated and embedded in paraffin. Sections (5 µm thick) were selected and stained with haematoxylin and eosin to observe histopathological changes microscopically (ECLIPSE TE 2000-U; Nikon Instruments Inc., Tokyo, Japan), with images acquired at 400× magnification.

### Western blot analysis

Total protein from the liver sample was extracted using the radioimmunoprecipitation assay buffer and proteinase inhibitor. Protein content was measured using the bicinchoninic acid protein assay kit (Nanjing Jiancheng Biotechnology Co., Ltd.). Sodium dodecyl sulfate–polyacrylamide gel electrophoresis was used to separate the protein samples, which were then transferred to a polyvinylidene fluoride membrane (Millipore Inc., Merck, Burlington, MA, USA) at 300 mA for 0.5 h before antibody treatment. The membranes were visualised and scanned using an enhanced chemiluminescence detection system from Alpha Innotech (part of Biosciences, San Leandro, CA, USA). Photoshop (Adobe Inc., San Jose, CA, USA) was used to process the images. ImageJ software v. 1.53 (*21*) was used to evaluate protein expression, with relative expression of the protein (REP) determined by normalizing the level of the target protein band (TPB) to that of the actin band (AB).


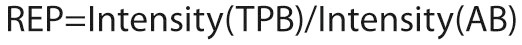
 /7/

### 16S rRNA gene amplification and MiSeq sequencing

Total genomic DNA was extracted using the DNA Extraction Kit (QIAGEN, Hilden, Germany) according to the manufacturer’s instructions. Polymerase chain reaction was performed using barcoded primers and Takara Ex Taq (Takara Bio, Inc., Otsu, Japan). MiSeq sequencing of intestinal flora was conducted by Shanghai Meiji Biomedical Technology Co., Ltd. (Shanghai, PR China). The raw sequencing data were preprocessed using Trimmomatic software (*22*), and QIIME software v. 1.8.0 (*23*) was then used for further analysis. Primer sequences were removed, and valid tags were clustered into operational taxonomic units (OTUs) with 97 % similarity using Vsearch software (*24*).

### Statistical analysis

Data are presented as mean value±standard deviation. The threshold for statistical significance was established at p<0.05. Statistical significance was assessed by one-way analysis of variance (ANOVA) using GraphPad Prism v. 8.0 (*25*).

## RESULTS AND DISCUSSION

### Effect of LSP156 on injury markers and histopathological examination

**Fig. 1:**
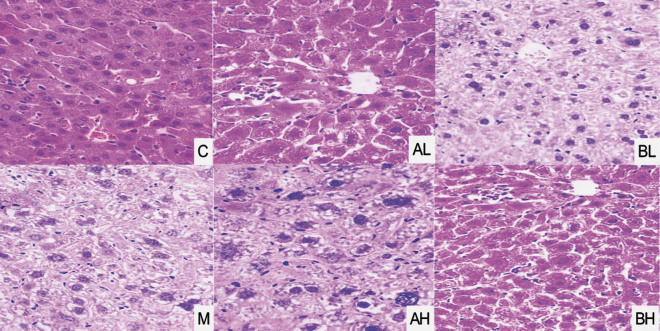
The stages of hepatic steatosis, hepatocyte morphology and inflammatory cell infiltration were checked, in six differently treated (with N-nitrosodiethylamine (NDEA) and *Lachnum* YM156 (LPS156)) mice groups, by hematoxylin and eosin (H&E) staining (400×). C=vehicle control, M=*w*(NDEA)=100 mg/kg, AL=*w*(NDEA)=100 mg/kg+*w*(LSP156)=50 mg/kg×4 weeks, AH=*w*(NDEA)=100 mg/kg+*w*(LSP156)=100 mg/kg×4 weeks, BL=*w*(NDEA)=100 mg/kg×4 weeks followed by *w*(LSP156)=50 mg/kg×4 weeks, and BH=*w*(NDEA)=100 mg/kg×4 weeks followed by *w*(LSP156)=100 mg/kg×4 weeks

Table 1 shows that the activities of alanine aminotransferase (ALT), aspartate transaminase (AST) and alkaline phosphatase (AKP), as well as the concentration of total bilirubin (TBiL) were significantly increased in N-nitrosodiethylamine (NDEA)-induced animals (p<0.05) compared to group C, whereas *Lachnum* YM156 (LSP156) therapy markedly reduced the levels of these liver injury indicators (p<0.05). Elevated hepatic transaminases (ALT and AST) and cholestatic markers (AKP and TBiL) quantitatively reflected the progression of hepatocellular damage, consistent with established hepatotoxicity models. NDEA-induced hepatotoxicity was associated with histopathological changes, including lobular inflammation, bridging fibrosis and steatonecrotic changes (*26*). These hepatic enzymes are established biomarkers of hepatic dysfunction, with increased activities directly indicating disrupted membrane integrity and subsequent necrosis during the pathophysiological continuum (*27*). Liu *et al.* (*28*) reported that Tuocha polyphenols could reduce the activities of ALT, AST and AKP, as well as blood lipid levels. LSP156 also had a positive and effective role in liver injury. Additionally, morphological changes in the liver tissue sections of the model group, such as nuclear hypertrophy, lipid vacuoles, inflammatory cell infiltration and lobular disarray, were observed, suggesting that treatment with NDEA can induce significant hepatic cellular damage. Furthermore, sinus dilatation, eosinophilic bodies, localised necrosis and several nuclear densifications were identified, whereas these injuries were alleviated in LSP156-treated mice (Fig. 1). The hepatotoxicity of NDEA in ICR mice was confirmed by assessing the biomarkers indicating histological abnormalities, hepatocyte necrosis and inflammatory responses, while the extent of liver damage was further supported by organ indices and oxidative damage (*26*, *28*).

**Table 1:** Effects of *Lachnum* YM156 (LPS156) on the values of alanine aminotransferase (ALT), aspartate transaminase (AST), alkaline phosphatase (AKP) and total bilirubin (TBiL)

Parameter	C	M	AL	AH	BL	BH
Activity (AST)/(U/g)	(6.3±1.2)^b^	(13.6±3.7)^a^	10.0±2.7	(7.5±1.1)^b^	12.4±2.6	9.6±1.1
Activity (ALT)/(U/g)	(18.2±1.9)^b^	(30.7±2.6)^a^	(28.8±1.8)^a^	(26.8±3.2)^a^	(27.6±1.3)^a^	(22.2±2.8)^b^
Activity (AKP)/(U/g)	(103±12)^b^	(580±11)^a^	(396±11)^ab^	(257±10)^ab^	(415±11)^ab^	(196.6±5)^ab^
*c*(TBil)/(μmol/L)	(1.1±0.1)^b^	(7.5±0.7)^a^	(6.6±0.4)^a^	(3.8±0.5)^b^	(3.9±0.6)^b^	(2.9±0.6)^b^

Data are presented as mean value±S.D., *N*=8. ^a^p<0.05 compared with the control group, and ^b^p<0.05 compared with the model group. C=vehicle control, M=*w*(NDEA)=100 mg/kg, AL=*w*(NDEA)=100 mg/kg+*w*(LSP156)=50 mg/kg×4 weeks, AH=*w*(NDEA)=100 mg/kg+*w*(LSP156)=100 mg/kg×4 weeks, BL=*w*(NDEA)=100 mg/kg×4 weeks followed by *w*(LSP156)=50 mg/kg×4 weeks, and BH=*w*(NDEA)=100 mg/kg×4 weeks followed by *w*(LSP156)=100 mg/kg×4 weeks

### Effects of LSP156 on viscera indices and oxidative stress markers

Exposure to NDEA induced hepatotoxicity and carcinogenic potential in mice (*8*). Its systemic effects were assessed by studying mass gain, organ index and oxidative damage in the model group, which differed from those in the normal group (Table 2). The mean body mass gain of NDEA-treated mice was (5.3±2.4) g, compared with (12.1±1.2) g in control mice. Treatments in the AL, AH, BL and BH groups improved the mass gain and organ index in liver injury mice. LSP156 slightly reduced the liver index in both low- and high-dose groups (AL, AH, BL and BH); however, no significant difference (p>0.05) was observed. LSP156 had no significant effect on the renal index. The spleen indices of the AL and AH groups were marginally higher than those of the model group, while the BL and BH groups remained relatively stable. Another source of NDEA-induced liver damage is a shift in antioxidant enzyme activities and antioxidant capacity. Compared with the control group, the activities of superoxide dismutase (SOD), catalase (CAT) and glutathione (GSH) in the NDEA group decreased by 17, 27 and 30 %, respectively, while the concentration of malonaldehyde (MDA) was significantly increased (Table 2). After LSP156 treatments, either as a dietary supplement or as a therapeutic drug, the activities of SOD, CAT and GSH increased, while concentration of MDA decreased. The activities of SOD and MDA in the BH group showed marked changes, indicating the best oxidative stress effects among the groups. These results indicate that LSP156 can enhance antioxidant activity by improving body mass and immune organ index, as well as having a significant regulatory effect on antioxidant capacity. Therefore, we believe that the anti-inflammatory and antioxidant activity of LSP156 is important in mice with liver injury. NDEA, a hepatocarcinogenic compound, induces oxidative imbalance through reactive oxygen species (ROS) overproduction, triggering hepatic injury and inflammatory cascades (*29*, *30*). Compromised antioxidant defences enable ROS accumulation beyond cellular neutralisation capacity, initiating DNA damage, membrane disruption and lipid peroxidation *via* oxidative stress-mediated pathways (*31*, *32*).

**Table 2:** Effects of *Lachnum* YM156 (LPS156) on viscera indices and oxidative stress parameters

Parameter	C	M	AL	AH	BL	BH
*m*(gain)/g	12.1±1.2	(5.3±2.4)^a^	(6.5±1.9)^a^	(7.7±2.7)^ab^	(6.0±1.3)^a^	(7.3±2.1)^ab^
Liver index/(mg/g)	6.0±0.5	5.8±0.7	5.3±0.8	(4.9±0.7)^a^	(4.5±1.4)^ab^	(4.9±1.0)^a^
Kidney index/(mg/g)	1.5±0.2	1.3±0.2	1.4±0.3	1.3±0.1	1.3±0.1	1.3±0.2
Spleen index/(mg/g)	0.3±0.1	0.4±0.1	0.5±0.1	0.4±0.1	0.3±0.1	0.3±0.1
Activity (SOD)/(U/mg)	1038±15	(860±7)^a^	(957±16)^b^	(998±14)^b^	(1083±15)^b^	(1123±28)^b^
Activity (CAT)/(U/mg)	37.6±2.6	(27.4±1.8)^a^	(29.3±2.1)^a^	(29.5±3.8)^a^	(28.1±2.7)^a^	30.0±3.9
*b*(MDA)/(nmol/mg)	3.2±0.5	(6.2±0.8)^a^	(4.5±0.4)^ab^	(3.3±0.3)^b^	(4.7±0.2)^ab^	(2.7±0.1)^b^
*b*(GSH)/(μmol/g)	6.5±1.4	(4.5±1.2)^a^	(5.0±1.1)^a^	(5.5±1.2)^b^	(4.4±1.0)^a^	(5.8±0.6)^b^

Data are presented as mean value±S.D., *N*=8. ^a^p<0.05 compared with the control group, and ^b^p<0.05 compared with the model group. C=vehicle control, M=*w*(NDEA)=100 mg/kg, AL=*w*(NDEA)=100 mg/kg+*w*(LSP156)=50 mg/kg×4 weeks, AH=*w*(NDEA)=100 mg/kg+*w*(LSP156)=100 mg/kg×4 weeks, BL=*w*(NDEA)=100 mg/kg×4 weeks followed by *w*(LSP156)=50 mg/kg×4 weeks, and BH=*w*(NDEA)=100 mg/kg×4 weeks followed by *w*(LSP156)=100 mg/kg×4 weeks

### Effect of LSP156 on the regulation of the inflammatory signalling pathway

**Fig. 2:**
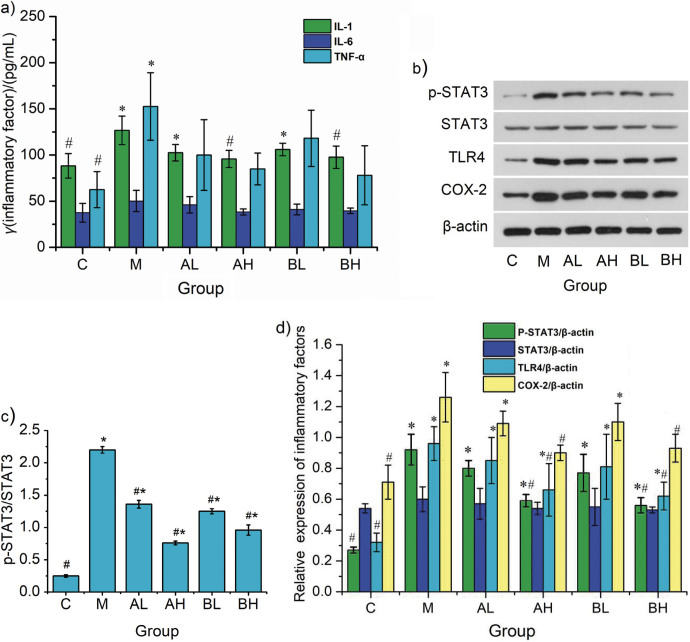
Effect of *Lachnum* YM156 (LPS156) on inflammatory factors and key proteins of the STAT3/COX-2 signalling pathway in N-nitrosodiethylamine (NDEA)-induced hepatic injury in mice: a) the concentrations of inflammatory markers IL-6 and TNF-α in serum were measured, b) protein expression of p-STAT3, STAT3, TLR4 and COX-2 in liver tissues, c) protein abundance of p-STAT3/STAT3 in liver tissues, showing the degree of activation of STAT3, and d) expression of inflammatory factor proteins relative to β-actin in liver tissue. Data are presented as mean value±S.D. with *N*=8 for results in a) and 3 in d). *p<0.05 compared with the control group, and ^#^p<0.05 compared with the model group. IL-6=interleukin-6, IL-1=interleukin-1, TNF-α=tumour necrosis factor-α, p-STAT3=phospho-signal transducer and activator of transcription 3, STAT3=signal transducer and activator of transcription 3, TLR4=toll-like receptor 4, COX-2=cyclooxygenase-2. C=vehicle control, M=*w*(NDEA)=100 mg/kg, AL=*w*(NDEA)=100 mg/kg+*w*(LSP156)=50 mg/kg×4 weeks, AH=*w*(NDEA)=100 mg/kg+*w*(LSP156)=100 mg/kg×4 weeks, BL=*w*(NDEA)=100 mg/kg×4 weeks followed by *w*(LSP156)=50 mg/kg×4 weeks, and BH=*w*(NDEA)=100 mg/kg×4 weeks followed by *w*(LSP156)=100 mg/kg×4 weeks

Serum TNF-α, IL-1 and IL-6 were quantified to assess inflammatory involvement in NDEA-induced hepatotoxicity and the hepatoprotective efficacy of LSP156. Fig. 2a shows a marked increase in these cytokines in group M compared to controls (C), with significant attenuation after the LSP156 treatment, suggesting partial resolution of hepatic inflammation. Mechanistic investigation of the therapeutic potential of LSP156 against NDEA-mediated liver injury showed altered phosphorylation status and expression of STAT3/COX-2 axis mediators (cyclooxygenase-2 [COX-2], STAT3 and TLR4) by Western blotting. STAT3 expression was similar in all groups, as shown in Fig. 2b and Fig. 2c, while the expression of p-STAT3, COX-2 and TLR4 in group M was increased compared with the normal group. Correspondingly, p-STAT3/STAT3 showed a similar pattern. However, after the treatment with LSP156, expression of p-STAT3/STAT3 decreased to varying degrees, particularly in the AH and BH groups (Fig. 2d). STAT3, a transcription factor, is activated by numerous cytokines, growth factors and proinflammatory cytokines, and plays an important role in the cell cycle, tumour development and hepatic inflammation (*33*-*35*). NDEA was found to enhance the amounts of TNF-α and IL-6 in ICR mice, thereby accelerating phosphorylation of STAT3 (*36*). Activated STAT3 interacts with or collaborates with nuclear EGFR at the COX-2 promoter region, causing enhanced COX-2 expression, which is linked to inflammatory responses and cancer (*37*). A previous study reported that TLR4 activation may regulate the COX-2/PGE2/STAT3 loop in hepatocellular carcinoma (HCC) cells (*38*). LSP156, as shown by Western blotting, reduces COX-2 protein levels by blocking STAT3 activation and TLR4 expression, helping to protect the liver and prevent damage.

### Effect of LSP156 on gut microbiota in NDEA-induced mice

**Fig. 3:**
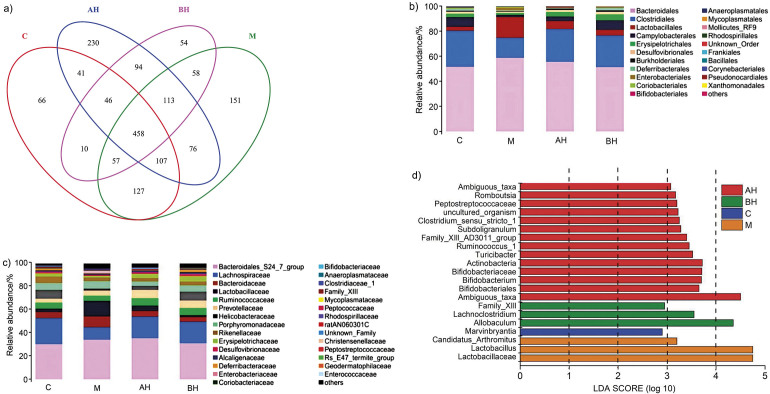
*Lachnum* YM156 (LPS156) changed the types and variety of gut bacteria in mice with liver damage caused by N-nitrosodiethylamine (NDEA): a) Venn diagrams showing the unique and shared operational taxonomic units (OTUs) in the gut microbiota of the respective groups. The presented values are the mean value±S.D., *N*=5, b) a heat map indicating the relative abundance distributions of each group at the order, and c) family levels of the top 30 (mean value±S.D., *N*=5), and d) LEfSe analysis showing the relative abundance among the different groups in mice. C=vehicle control, M=*w*(NDEA)=100 mg/kg, AL=*w*(NDEA)=100 mg/kg+*w*(LSP156)=50 mg/kg×4 weeks, AH=*w*(NDEA)=100 mg/kg+*w*(LSP156)=100 mg/kg×4 weeks, BL=*w*(NDEA)=100 mg/kg×4 weeks followed by *w*(LSP156)=50 mg/kg×4 weeks, and BH=*w*(NDEA)=100 mg/kg×4 weeks followed by *w*(LSP156)=100 mg/kg×4 weeks

The liver, as the organ most affected by the gut microbiota, is increasingly regarded as a crucial and promising therapeutic target for several complex disorders, especially HCC (*39*). Numerous studies have shown that polyphenols serve as alternative agents for modulating the gut microbiota (*40*). We examined the impact of LPS156 on the gut microbiota by assessing the intestinal microbial population structure using gene sequencing. Fig. 3a shows Venn diagrams that compare the diversity of gut microbes in the two more effective treatment groups (AH and BH), the control group (C) and the model group (M).

The total number of operational taxonomic units (OTUs) in the model group increased compared with the control group, with more specific OUTs present. Furthermore, the OTUs in the treatment groups (AH and BH) overlapped more with group C than with group M. Based on the relative abundance of intestinal flora among the different groups shown in Fig. 3b and Fig. 3c, the species composition of groups M and C differed. Additionally, *Bacteroidales* and *Clostridiales* were the predominant orders, with the abundance of *Bacteroidales* and *Lactobacillales* in the model group significantly exceeding that in group C. The abundances of *Bacteroidales*, *Lactobacillales* and *Enterobacteriales* in group M were higher than in group C. In contrast to group C, *Clostridiales*, *Campylobacterales*, *Erysipelotrichales* and *Deferribacterales* showed reduced abundance. LPS156 treatment significantly decreased the populations of *Bacteroidales* (AH: 3.2 %, BH: 4.2 %) and *Lactobacillales* (AH: 10.2 %, BH: 1.9 %) (Fig. 3b). Notably, LPS156 restored these reduced orders to control levels, demonstrating its regulatory effect on gut microbiota homeostasis. Taxonomic analysis revealed differential abundance of seven families (*Lachnospiraceae, Bacteroidaceae, Lactobacillaceae, Ruminococcaceae, Prevotellaceae, Helicobacteraceae* and *Porphyromonadaceae*) compared to controls. NDEA intervention specifically reduced key families, including *Lachnospiraceae* (11.7 %), *Helicobacteraceae* (5.9 %) and *Rikenellaceae* (2.5 %) at the family level (Fig. 3c). Linear discriminant analysis Effect Size (LEfSe) identified taxonomic distinctions: *Lactobacillus* and *Arthromitus* dominated group M, whereas *Marvinbryantia* characterised group C. The AH group showed greater microbial diversity than BH, predominantly comprising *Bifidobacteriaceae*, *Actinobacteria* and *Turicibacter* (Fig. 3d). Polyphenols reportedly enhanced *Lachnospiraceae*, *Clostridiales*, *Campylobacterales*, *Erysipelotrichales* and *Deferribacterales* - taxa associated with the mitigation of gastrointestinal and hepatic inflammation, including fibrotic progression (*41*). In accordance, greater abundance of Bacteroidetes observed in murine models (in line with our data) may represent a risk factor for endogenous infections or colorectal carcinogenesis (*42*).

### Metabolic pathway of the gut microbiota

**Fig. 4:**
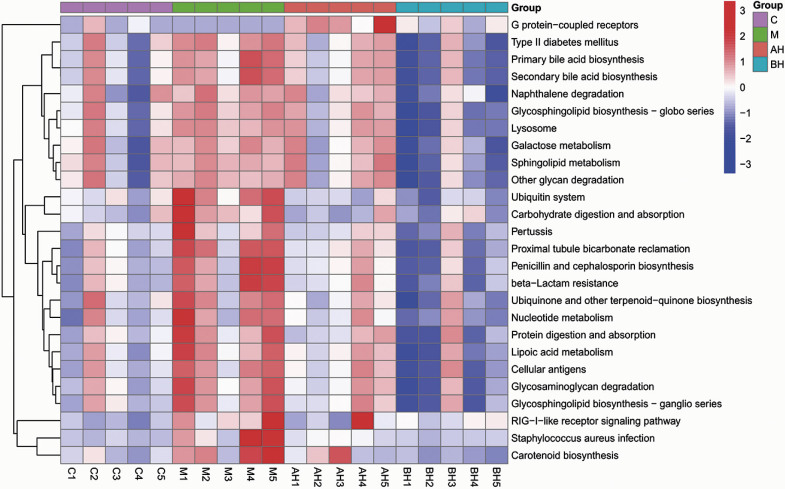
The predicted difference in the KEGG metabolic pathways of the gut microbiota from different groups: C=vehicle control, M=*w*(NDEA)=100 mg/kg, AL=*w*(NDEA)=100 mg/kg+*w*(LSP156)=50 mg/kg×4 weeks, AH=*w*(NDEA)=100 mg/kg+*w*(LSP156)=100 mg/kg×4 weeks, BL=*w*(NDEA)=100 mg/kg×4 weeks followed by *w*(LSP156)=50 mg/kg×4 weeks, and BH=*w*(NDEA)=100 mg/kg×4 weeks followed by *w*(LSP156)=100 mg/kg×4 weeks

Functional genomic profiling using the Kyoto Encyclopedia of Genes and Genomes (KEGG) pathway analysis revealed significant NDEA-induced perturbations in microbial metabolic pathways, particularly in carbohydrate metabolism, terpenoid-quinone biosynthesis and proteolytic processing (Fig. 4). Comparative analysis demonstrated that LSP156 had greater therapeutic efficacy than prophylactic effects in mitigating NDEA-induced hepatotoxicity, with pathway restoration correlating with injury attenuation.

### Associations between the phenotype and gut microbial community

**Fig. 5:**
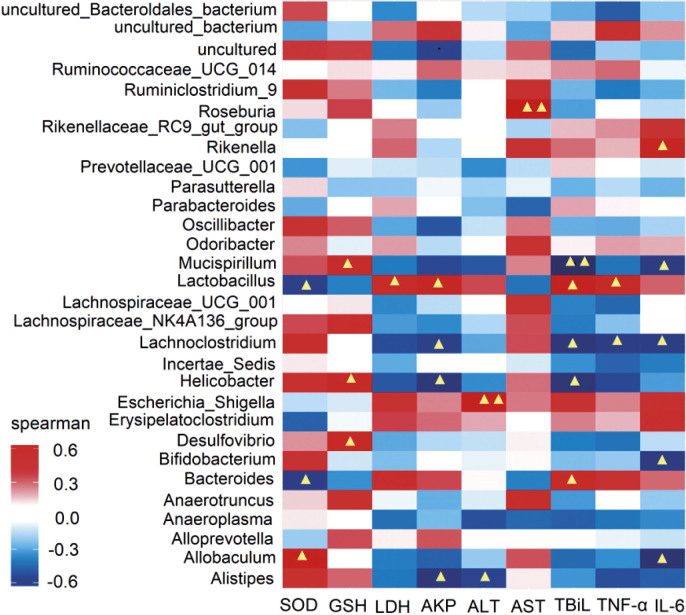
Heatmap of the correlations between metagenomic genera and phenotype. ^▲^ indicates a significant correlation at p<0.05, ^▲▲^ indicates a significant correlation at p<0.01. The correlation was determined by the Spearman index. SOD=superoxide dismutase, GSH=glutathione, LDH=lactate dehydrogenase, AKP=alkaline phosphatase, ALT=alanine aminotransferase, AST=aspartate transaminase, TBiL=total bilirubin, TNF-α=tumour necrosis factor-α, IL-6=interleukin-6

Correlation analysis of the top 30 gut microbiota with host parameters revealed significant associations between microbial taxa and oxidative, hepatic and inflammatory markers (Fig. 5). Oxidative stress markers showed that *Ruminiclostridium*, *Roseburia*, *Oscillibacter*, *Mucispirillum*, *Lachnospiraceae* NK4A136 group, *Helicobacter* and *Alistipes* were positively correlated with SOD and GSH levels, while lactate dehydrogenase (LDH) was inversely associated. Hepatic function indices demonstrated positive correlations with *Ruminococcaceae* and *Escherichia shigella*, while *Anaeroplasma* was negatively associated with ALT, AST, AKP and TBiL (p<0.05). Notably, *Lachnoclostridium* had anti-inflammatory properties through negative correlations with TNF-α and IL-6. *Bacteroides* and *Lactobacillus* maintained positive correlations with proinflammatory hepatic indicators (LDH, AKP, TBiL, TNF-α and IL-6) despite overall microbiota divergence. Pathophysiological correlation mapping further revealed a significant association between *Bacteroidetes* and TNF-α/IL-6 (p<0.05), suggesting that this phylum mediates inflammatory cascades in NDEA-induced hepatotoxicity through both direct and indirect pathways. Lactobacillaceae showed positive correlations with hepatic injury biomarkers (AKP, ALT and TBiL) and proinflammatory cytokines, suggesting that immune-mediated pathways promote its beneficial microbial expansion. LSP156 supplementation attenuated NDEA-induced hepatotoxicity by restoring microbial homeostasis, highlighting the modulation of the gut–liver axis as its protective mechanism (*43*).

## CONCLUSIONS

Polyphenolic compounds mitigate hepatopathology by reducing oxidative stress, modulating anti-inflammatory signalling and altering the gut–liver axis. Our results show that *Lachnum* YM156 (LSP156) increases body mass and organ indices, reduces oxidative damage and inflammatory responses, alleviates liver tissue injury and regulates the STAT3/COX-2 signalling pathway in N-nitrosodiethylamine (NDEA)-induced liver injury mice. Furthermore, LSP156 alleviates gut microbiota dysbiosis and adjusts microbial composition to reduce liver damage. This study highlights the connection between STAT3/COX-2 signalling and gut microbiota alterations, providing a comprehensive therapeutic approach that could significantly influence the management of liver diseases.
